# Efficacy of Laser Therapy in Trigeminal Neuralgia: a Systematic Review

**DOI:** 10.30476/dentjods.2023.95758.1889

**Published:** 2024-03-01

**Authors:** Sara Haghighat, Fahimeh Rezazadeh, Hossein Sedarat, Amir Tabesh, Elham Tayebi Khorami, Kiana Aghasadeghi

**Affiliations:** 1 Student Research Committee, School of Dentistry, Shiraz University of Medical Sciences, Shiraz, Iran; 2 Oral and Dental Disease Research Center, Dept. of Oral and Maxillofacial Medicine, School of Dentistry, Shiraz University of Medical Sciences, Shiraz, Iran; 3 Undergraduate Medical Student, Student Research Committee, Jahrom University of Medical Sciences, Jahrom, Iran; 4 Undergraduate Student, Student Research Committee, School of Dentistry, Shiraz University of Medical Sciences, Shiraz, Iran; 5 Undergraduate Student, Student Research Committee, School of Dentistry, Azad University, Shiraz, Iran

**Keywords:** Trigeminal neuralgia, Laser therapy, Review systematic, Facial pain

## Abstract

**Statement of the Problem:**

Trigeminal neuralgia is the most common and disabling type of neuralgia in craniofacial region. Because of adverse effects of first and second lines of treatment, new modalities including laser therapy have been investigated for treatment of trigeminal neuralgia.

**Purpose:**

The aim of this study was to review the effect of laser in trigeminal neuralgia. PubMed, Scopus, Web of Science, Science Direct, and Embase databases from December 1983 to August 2020 were searched using keywords “trigeminal neuralgia” and “laser”. Our inclusion criteria were interventional studies with a randomized clinical trial design, which used laser for treatment of trigeminal neuralgia.

**Materials and Method:**

In this systematic review, a total of 269 records were identified through systematically searching aforementioned databases among which, 30 were from PubMed and 44 were from Web of Science. A total of 111 records were duplicated and were therefore removed.

**Results:**

Only 17 records were considered relevant after reading title and abstracts. After reading full texts of the articles, 13 met the eligibility criteria and were included in our review.

**Conclusion:**

This review revealed that low-level laser therapy reduces pain in trigeminal neuralgia specially diode lasers, although there are no standardized protocols for laser procedures.

## Introduction

Neuralgias are amongst the most painful situations that are experienced by human [ 1 ]. Trigeminal neuralgia (TN) is the most common and disabling type of neuralgia in craniofacial region [ 2 - 3 ] which is characterized by paroxysmal attacks of shock-like stubbing, penetrating, sharp pain that mostly happens unilateral in skin of eyebrows, eyes, lips, nose, scalp, forehead, jaw and periocular structures [ 1 , 4 ]. The duration of pain episodes can vary in each patient but they occur and terminate suddenly and are mostly less than two minutes [ 3 , 5 ]. There is usually at least one trigger point in affected patients and innocuous stimuli such as brushing, eating, talking, and even washing the face may cause onset of the pain [ 5 - 6 ].

 Different numbers have been reported as the prevalence of TN such as 1 in 25,000 [ 7 ], 4 out of 100000 [ 8 ], 1 in 15000 individuals [ 5 ], and 0.05% [ 9 ] but the actual prevalence might be significantly higher due to undiagnosed or misdiagnosed cases. Mostly females and middle-aged individuals older than 50 years old are involved with this disease [ 3 , 7 , 10 ]. The female to male ratio of TN is reported to be 3 to 1 [ 10 ]. 

There are different theories that explain the possible causes of paroxysmal pain in neuralgia pain episodes including external pressure of artery on trigeminal nerve root (neurovascular decompression), focal demyelination of trigeminal nerve afferents and hyper excitability of axons, brain stem infarction, cerebellopontine angle tumors and abnormality in expression of voltage-gated sodium channels [ 3 , 8 , 10 - 11 ]. However, the exact underlying mechanism of TN is not completely understood and is remained unclear yet.

The diagnosis and management of TN is particularly complicated and require a multi-disciplinary approach including neurology, neurosurgery, oral and maxillofacial surgery, and oral medicine specialists [ 7 ]. Although there are some tools for diagnosis of TN including laser evoked potentials (LEP) and magnetic resonance imaging (MRI) [ 12 - 13 ], none of them can provide a definitive diagnosis and still, a precise interview with the patient is the best method for ruling out other differential diagnoses [ 8 ].

There are different strategies for treatment of TN. Drug therapy by anti-epileptic drugs is the first line of treatment. Carbamazepine is the drug of choice according to several evidences [ 6 - 7 , 10 , 14 - 15 ]. However, drug therapy is approximately non-satisfying in 20-50% of cases [ 5 , 10 ] and its efficacy decreases over time [ 15 ]. Carbamazepine also has side effects like headache, dizziness, decrease in postural stability and alertness, nausea, erythema multiform, and decrease in white blood cell count that turns into aplastic anemia in severe cases [ 5 , 9 , 16 - 17 ] that further limits its use. 

Surgery is another more invasive treatment modality, which is indicated in more complicated refractory cases. There are several methods of surgery including stereostatic radiosurgery [ 3 , 6 , 18 ], ganglion block surgery [ 11 ], percutaneous radiofrequency thermal rhizotomy [ 7 ], and microvascular decompression [ 19 ]. Surgery is not acceptable by all patients and it should be postponed concerning its invasive nature and side effects. Paresthesia, dysesthesia, numbness of facial skin, and high rate of pain recurrence are among the most common side effects [ 11 , 20 ] but cerebrospinal fluid (CSF) leak, infarction, hematoma, aseptic meningitis, and hearing loss has been also reported as more complicated side effects [ 6 ]. 

Because of above adverse effects of first and second lines of treatment, new modalities have been investigated for treatment of TN including laser therapy and acupuncture [ 8 , 16 , 21 - 22 , 27 ]. Low level laser therapy (LLLT) has been used in treatment of different diseases especially chronic pains and has been reported as an effective method for alleviating pain [ 8 , 23 - 24 ]. Its mechanism of pain reducing action is through decreasing histamine, bradykinin, acetylcholine and prostaglandin E2 and increase in expression of endorphin mRNA precursor, ATP, and enkephalins [ 8 , 11 , 16 ].

Although few studies have evaluated laser therapy in treatment of TN, these studies reported controversial results and had different methodology; hence, in this systematic review we aim to review the effect of laser in treatment of TN.

### Search sources

We searched for articles, which evaluated the effect of laser on treatment of TN. In 26 August 2020, PubMed, Scopus, Web of Science, Science Direct and Embase databases were searched using keywords “trigeminal neuralgia” and “laser” in title and abstracts parts using advanced search. 

### Search strategies

Our search strategy was based on a PICO question as follows: “Does using laser improve treatment of trigeminal neuralgia?” our search strategy included two parts: laser and TN which were grouped by Boolean operator “AND”. Simple Keywords were selected in order to include all possible results. 

### Selection of studies

Our inclusion criteria were interventional studies from December 1983 to August 2020 with a randomized clinical trial design, which used laser for treatment of TN. Studies in languages other than English and animal model studies were excluded from our results. 

### Data collection

Reviewers independently evaluated studies and collected data about patients (total number of TN patients and demographic information of both case and control groups), characteristics of intervention (laser type, laser wavelength, sessions of laser therapy, time / dose of exposure), comparison (placebo, surgical methods or other methods for treatment of TN), follow up (duration of follow up) and outcome characteristics (assessment tool, values in case and control groups).

### Analysis of risk of bias in included studies

Two reviewers independently assessed risk of bias in all included studies according to the Cochrane Collaboration's tool for assessing risk of bias (chapter 8.5 of the Cochrane Handbook for Systematic Reviews of Interventions, 2011). 

### Analysis of data

Data is reported descriptively since the results were not eligible for quantitative analysis (Meta-analysis) according to statistics specialist consult. 

### Selection of studies

**Figure 1:**
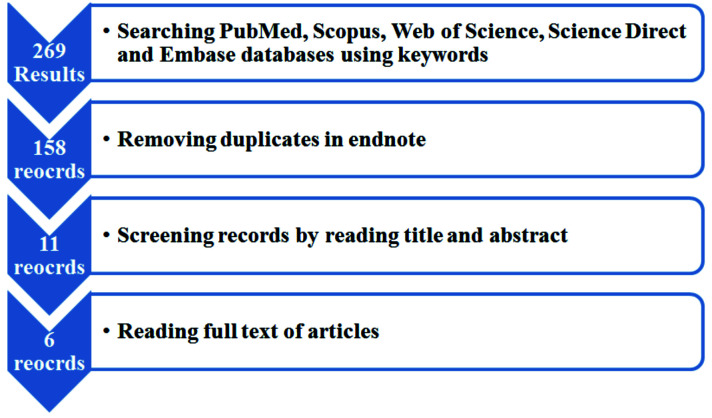
Flowchart of study

**Figure 2:**
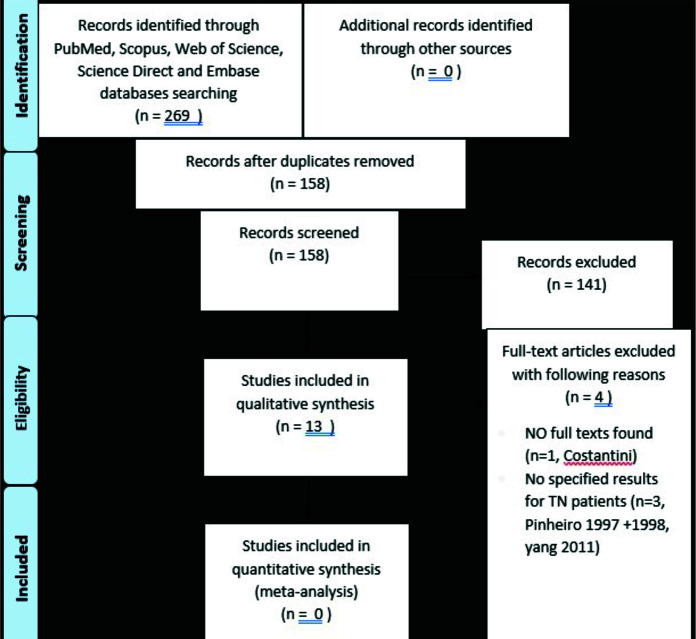
PRISMA 2009 Flow Diagram of study

A total of 269 records were identified through systematically searching aforementioned databases among which, 30 were from PubMed and 44 were from Web of Science. A total of 111 records were duplicated and were therefore removed. Only 17 records were considered relevant after reading title and abstracts. The main reasons for exclusion of studies were irrelevant methodology, types other than journal articles (such as conference abstracts, books, and so on) and articles in languages other than English. After reading full texts of the articles, 13 met the eligibility criteria and were included in our review. Running meta-analysis was not feasible regarding our statistics specialist consult. The process of selecting studies is shown in Figures 1 and 2.

### Included studies characteristics

The characteristics of included studies are summarized in Table 1. Two studies compared the effect of carbamazepine with and without laser therapy in treatment of TN patients [ 8 , 16 , 25 - 26 ]. One of the studies evaluated the adjunctive effect of laser therapy in patients undergoing ganglion block surgery [ 11 ]. In another studies, different types of laser wavelengths were compared [ 27 - 28 ]. The other articles compared laser therapy as the only treatment with sham laser in groups [ 29 - 30 , 32 - 33 ]. Only one study was compared laser with trans cranial electromagnetic (TES) [ 31 ]. In most of studies, response to treatment were evaluated by visual analogue scale (VAS) except for Walker study which used pain effect scale (PES) and 5-HIAA concentration which is an indicator of pain measured in urine samples [ 29 ]. Others were assessed pain by numerical rating scale (NRS) [ 26 , 28 , 30 - 31 ].

**Table 1:** The result of all reviewed article

	Laser type	Laser wavelength	Sessions of laser therapy	Comparison (Control group)	Number of TN patients (Case/ control)	Result assessment	Duration of Follow up	Time / dose of exposure	Result
Walker [ 33 ] 1983	He-Ne	632nm	30 (3 per week, 10 weeks)	Sham laser	12 (9/3)	### PES +5-HIAA (24 hour urine sample)	After 20 sessions of therapy	20 seconds/1mW (30 seconds increase in each week)	85 % estimated pain relief
Hensen *et al*. [ 29 ] 1990	IR	904 nm	8 (2 per week, 4 weeks)	Inactive placebo laser probe	1 was primary TN+3 secondary TN Patients	# VAS+5-HIAA (24 hour urine sample)	2,4,6 months	60 seconds/ Maximum dose of 4.7 J/cm^2^	NOT significant
Aghamohammadi *et al*. [ 35 ] 2012	IR	890 nm	12 (every other day)	Sham laser (Ganglion block in both groups)*	42 (21/21)	VAS	Days: 1,3,5,7	3-10 J per point	NOT significant
							Months: 1,3,6		
Amanat *et al*. [ 16 ] 2013	GaAs	980 nm	10 (3 per week)	Sham laser (carbamazepine in both groups)	26(12/14)	VAS	2,4 months	5 minutes on each tender point/12.73 J/ cm^2^	NOT significant
Antonic *et al*. [ 27 ] 2017	GaAlAs	810 nm	20 (5 per week, 4 weeks)	Two wavelength	20 (10/10)	VAS **	Immediately after treatment	10 minutes /30 mW, 3.0 J/cm^2^ ***	Significant (post vs pre) (810 nm vs 660 nm)
Ebrahimi *et al*. [ 8 ] 2018	GaAlAs	810 nm	9 (3 per week, 3 weeks)	Sham laser (carbamazepine in both groups)	30 (15/15)	VAS	1 month	25 seconds / 5J energy, max power 200 mW	significant
Ibrahim Saeda *et al*. [ 24 ] 2013	HeNe laser	830 nm	24 (3 per week, 8 weeks)	Laser/transcranial electromagnetic stimulation(10Hz)	30 (15/15) multiple sclerosis patients with TN	## NRS (0,5,10)	Immediately after treatment	Intra oral: 1-2 mins Extra oral: 10 mins/15 mW, density 150-170 mw/cm^2^	significant
Eckerdal *et al*. [ 25 ] 1996	GaAlAs	830 nm	5 (1 per week, 5 weeks)	Sham laser (analgesics consumption in both groups)	30 (14/16)	VAS	Immediately after treatment, after 1 year	32mW,laser density of 9.2 J/ cm^2^	Significant
Pinheiro *et al*. [ 28 ] 1998	IR 30 multilaser	830,632.8, 670 nm	14 (2 per week, 6 weeks and 2 sessions after one month)	3 wavelength	53	NRS	Immediately after treatment, 1 month later	40 mW, laser density of 3.9 (830 nm) to 0.2 (632.8 nm) to 0.8 (670 nm)	Significant
Somchai Sessirisombat 3[ 30 ] 2017	CO_2_ laser		1 session with CO_2_ laser	Group who refuse surgery and non -tolerable to drugs	36	NRS	1week,1monts, 3months, 6 months and 1 year	30seconds /Power 5 W	significant
Kim *et al*. [ 51 ] 2003	He-Ne, Ga-AI-As and CO_2_ lasers	904nm for Ga-AI-As 632nm for He-Ne	6	Laser group/ laser with carbamazepine	25	VAS		20mW for He- Ne and 40mW for Ga-Al-As 4 J/ cm^2^,100 Hz	significant
Intsar S. Waked *et al*. [ 32 ] 2015	He-Ne		24 (3 per week, 8 weeks)	3 groups, 2 groups with 2 methods of laser application, 1 group with placebo laser probe	45(15,15,15)	NRS	Immediately after treatment	15 minute	significant
Wichuda Kongsong *et al*. [ 26 ] 2020	CO_2_ laser		1 session	1 group, all use carbamazepine before and after	50	NRS	1week,1month,every 3-6 months	30seconds / Power 5 W	significant

* : all patients were diagnosed with refractory TN and were using carbamazepine at baseline.

** : Data of VAS were presented as median and (5th-95th) percentile boundaries.

**** : not considered as one of 6 final included studies.

*** : The treatment time (t) for each application point was calculated using the following equation: $\mathrm{t(sec)}=3.0\frac{1}{{\mathrm{cm}}^{2}}\times 1\frac{{\mathrm{cm}}^{2}}{0.003}\mathrm{(W)}$

# NRS: Numeric Rating Scale /

## VAS: visual analogue scale /

### PES: pain effect scale

Most (nine) of the studies have reported significant improvement in pain relief after using laser as treatment of TN [ 25 - 28 , 30 , 32 ]. Walker reported 85% pain relief in their study [ 39 ]. Only three studies have reported that there was no significant improvement in pain of TN patients after laser therapy [ 11 , 16 ].

### Assessment of risk of bias

The information about risk of bias of each article is summarized in Table 2. Only two articles was low risk in all domains [ 16 , 28 ] and the others had unclear risk of bias in at least one of the domains. In attrition bias domain, all the studies were low risk except two [ 27 , 30 ]. Only one study mentioned other possible sources of bias [ 33 ] although some studies had high risk concerning reporting bias [ 32 ].

**Table 2:** Risk of bias of eligible studies

	Random sequence generation (selection bias)	Allocation concealment (selection bias)	Blinding of participants and personnel (performance bias)	Blinding of outcome assessors (detection bias)	Incomplete outcome data addressed (attrition bias)	Selective outcome reporting (reporting bias)	Other bias
Walker [ 33 ] 1983	No randomization	Unclear	Low	Low	Low	Low	Sampling bias
Hensen *et al*. [ 29 ] 1990	Low	Unclear	Low	Low	Low	Low	Unclear
Aghamohammadi *et al*. [ 35 ] 2012	Low	Low	Unclear	Unclear	Low	Low	Unclear
Amanat *et al*. [ 16 ] 2013	Low	Low	Low	Low	Low	Low	Unclear
Antonic *et al*. [ 27 ] 2017	Unclear	Unclear	Unclear	Unclear	Unclear	High	Unclear
Ebrahimi *et al*. [ 8 ] 2018	Low	Low	Unclear	Unclear	Low	High	Unclear
Ibrahim Saeda *et al*. [ 24 ] 2013	Low	Unclear	Low	Low	Low	Low	Unclear
Eckerdal *et al*. [ 25 ] 1996	Low	Low	Unclear	Unclear	Low	Low	Unclear
Pinheiro *et al*. [ 28 ] 1998	Low	Low	Low	Low	Low	Low	Unclear
Somchai Sessirisombat [ 30 ] 2017	Unclear	Unclear	Low	Low	Unclear	High	Unclear
Kim *et al*. [ 51 ] 2003	Low	Unclear	Low	Low	Low	Low	Unclear
Intsar S. Waked *et al*. [ 32 ] 2015	Low	Low	Unclear	Unclear	Low	High	Unclear
Wichuda Kongsong *et al*. [ 26 ] 2020	Low	Low	Unclear	Unclear	Low	Low	Unclear

## Discussion

Although most assessed studies have demonstrated significant pain reduction of chronic orofacial pain by LLLT [ 8 , 24 , 27 , 34 ], some others did not report significant pain relief after laser therapy [ 16 , 35 ]. In this systematic review, we assessed the effect of laser therapy in treatment of primary TN. After searching was completed, only three studies were found that compared laser with placebo (in order to eliminate the placebo effect) for TN pain control [ 29 - 32 ]. Other studies compared laser with other modalities such as medicine, surgery, ganglion block, TES, or compared two different wavelengths of laser with each other [ 8 , 11 , 27 , 36 ]. Furthermore, some studies used laser for secondary TN or multiple sclerosis patients [ 29 ]. Based on statistician opinion, meta-analysis was not indicated due to non-uniform (equivalent) data of studies. Therefore, we only reviewed 13 similar articles based on effect of laser on TN pain reduction. 

A total of 9 of 13 reviewed articles reported a significant pain decrease in TN [ 8 , 27 ]. Walker also reported 85% pain relief after laser therapy [ 36 ], although others pointed no significant difference between laser and sham laser group [ 8 , 11 , 16 ]. Comparison the result of different studies is difficult due to variety in patient selection, number of follow up sessions, dose of laser therapy (power or time), and type of laser (wavelengths). However, in all of these articles, pain reduction, whether significant or not, may be related to psychological effects (placebo effect) [ 29 ] In most of reviewed studies, LLLT was used except two studies that employed CO_2_ laser [ 26 , 30 ].

Low-level lasers have super luminous diodes and a mixture of infrared and red photons in the shape of laser. The patient feels no pain and this kind of treatment is not invasive at all. These lasers are low power in comparison with high-power ones such as surgical lasers. While the laser beam is released, the level of adenosine triphosphate in cells grows higher by light absorption of cytochrome c oxidase in the mitochondria [ 37 ].

In previous researches, LLLT has been used for the treatment of nerve problems. These studies have shown increased nerve function and improved capacity for myelin production [ 38 , 39 ]. Induction of analgesic effects has been shown by LLLT. Pain relief is the result the increase in serotonin and endorphin levels in combination with the decrease in prostaglandin E2 and bradykinin levels. The reduction in pro-inflammatory factors (cytokines) such as tumor necrosis factor alpha (TNFα), interleukin (IL)-1b, and the enlargement in amount of anti-inflammatory cytokines such as IL-10, quickly alleviates the inflammation [ 37 ].

In an animal study, researchers showed laser stimulated axonal growth in injured nerves and it increased the change of PGG2 and PGH2 into PG12 (prostacyclin) that act in vasodilation and anti-inflammatory action [ 38 , 40 ]. The other mechanism of pain reduction in LLLT is blocking pain transmission in the peripheral nervous system by generating varicose veins which results in a reduction in fast axonal flow speed [ 41 ]. Moreover, the change in pain threshold and stimulating the neurogenesis has been suggested [ 42 ]. Therefore, LLLT by all of these mechanisms can result in pain control in TN patients. 

It is substantial to choose the most appropriate wavelength while using laser therapy for each certain disease. Wavelength of laser directly determines the degree of penetration through the tissues. A wavelength of 830nm, capable of reaching the cortical and alveolar bone tissues, is considered to have the deepest penetration and yet more effectiveness than wavelengths between 620 and 670 nm [ 43 ]. Red and infrared lasers are efficient for different conditions. Red laser is indicated for superficial injuries regarding its weak penetration and therefore greater absorption while passing through the tissues [ 44 ]. Infrared laser, on the other hand, is indicated for causing instant and impermanent analgesic effects, following from its deeper penetration through biological membranes. It interacts with the polarity alterations and induces analgesia by causing hyperpolarization on cell membrane (light-cell biological interaction) which is known to be a photo-physical mechanism [ 41 ]. Among the included studies, 4 of 5 used diode laser such as Ga-Al-As or Ga-As [ 8 , 11 , 16 , 27 ], that showed effective for decreasing pain. Nonetheless, the diode laser with wavelength of 660-980 nm was used widely for treatment of TN and usually provided good results; it seems that this type of laser might be better for pain control of TN [ 5 ].

It should be mentioned that definitive diagnosis of TN is the most important part of treatment plan, because other chronic orofacial pain sources may interfere with TN treatment results [ 45 ].

Regarding the traits of the studies, it is highly of note that, although the studies point out the results shortly after the beginning of laser therapy, there is no agreement about the protocol of laser application. 

With regard to the number of laser application, studies applied 9 up to 30 days of treatment. Moreover, frequency of sessions usually varied between 2-3 times per week, although, one study used laser in continuous days for patients [ 27 ]. The interval sessions for laser therapy result in decreasing the cumulative effect of laser in tissue, which induce the inhibitory effect or may exacerbate the pain. 

The parameters between the studies are of great variety and differ concerning recommended dose and time of application. Due to the lack of coordination between the mentioned parameters, and also the duration and number of the sessions, it is difficult to approve a certain protocol for pain control in TN. 

One of the common challenges in laser therapy is the variation of the treatment dose (power density) between the studies. In our review, the applied dose was ranged between 3 up to 12j/cm^2^. Based on Arndt-Schulz curve (rule) for LLLT, optimal dose should be in range of 0.001-10 j/cm^2^ for stimulating physiologic process and higher dose result in inhibitory effect [ 46 ]. However, some stated that for pain control, we need inhibitory effect [ 47 ].

In our review, the risk of bias analysis in most of the studies was low; however, the obtained information from some domains of evaluation was insufficient. 

Articles with language other than English were excluded from our review because they were not reachable. Furthermore, case reports also were not included to our study. 

Excluding the previously mentioned articles can be pointed at as a possible limitation of this review study. However, because of English being considered the language of science, it has been claimed that the exclusion of non-English articles does not seemingly bias systematic reviews [ 53 ]. Regarding the databases, we attempted to reduce the incident of bias as much as possible, by involving articles from other sources as well, including gray literature, on account of inadequate amount of studies. It is unfortunate to note that, this research was not able to add any new articles to this review.

Thus, to conduct a sophisticated future research, various parameters of laser therapy and favorable results of previous studies must be in order to establish an irradiation protocol for TN management. Besides, it should be considered that some outcomes are patient-dependent, especially the financial aspect of this therapy in order to compare the cost-effectiveness of this procedure with the conventional therapy.

## Conclusion

This review study showed that laser therapy, especially diode laser (LLLT), might be beneficial in managing TN patients. Despite the fact that there is no standard method for laser treatment, sufficient data to solve this issue is yet to be discovered and placebo effect may influence the pain reduction.
